# Splenic rupture after sexual intercourse in a pregnant woman: an extremely rare case

**DOI:** 10.2144/fsoa-2021-0027

**Published:** 2021-06-22

**Authors:** Abdallah Chaar, Wael Abdallah, Richard Kharrat, Malek Nassar

**Affiliations:** 1 Department of Gynecology & Obstetrics, Hôtel-Dieu de France University Hospital, Saint Joseph University, Beirut, Lebanon; 2 Department of Gynecology & Obstetrics, Bellevue Medical Center Hospital, Saint Joseph University, Beirut, Lebanon

**Keywords:** abdominal pain, pregnancy, spleen rupture, trauma

## Abstract

A 33-year-old pregnant woman presented at 36 weeks gestation to the emergency with acute abdominal pain that started after vaginal intercourse. No bruising was present on the abdominal examination. An emergent cesarean delivery was performed for resistant hypotension and collapse. A fetus with cardiac arrest was delivered, and active spleen bleeding was identified at the splenocolic and gastrosplenic ligament insertion. The patient had a conservative treatment of the spleen and an uncomplicated postoperative course. The infant was resuscitated and discharged after 18 days. In conclusion, traumatic spleen rupture is an etiology to consider in pregnant women presenting with acute abdominal pain following sexual intercourse. Early suspicion and emergent cesarean delivery are the keys to optimize maternal and perinatal outcomes.

## Lay abstract

Although sexual activity is generally considered safe in pregnancy, we present a rare case of post-coital spleen rupture in a third-trimester pregnant woman. However, rupture of the spleen is an unusual but serious cause of acute abdominal pain in pregnancy and is associated with both maternal and fetal morbidity. The aim of this article is to highlight the role of early suspicion to optimize maternal and perinatal outcomes.

A ruptured spleen is a serious emergency that occurred after a blunt trauma of the abdomen or after an evolution of a splenomegaly [1]. It carries a mortality of around 12% [2]. However, rupture of the spleen is an unusual but serious cause of acute abdominal pain in pregnancy and is associated with both maternal and fetal morbidity. This pathology can be classified as traumatic or spontaneous, which commonly occurs in a pre-existing pathology of the spleen. However, by reviewing the literature, there are 35 case reports that described a splenic rupture in a pregnant woman after trauma, but just one case report presented a spontaneous rupture of a normal spleen [3–5].

We are reporting here a rare case of post-coital spleen rupture in a third-trimester pregnant woman.

## Case report

A 33-year-old married woman, *gravida* 4 *para* 3 (three previous caesarean sections), with no relevant medical or obstetrical history, presented to the emergency room at 36 weeks of pregnancy with severe abdominal pain. Until that point, the course of the pregnancy was uneventful without any history of abnormal placentation or hypertensive disorder.

The patient complained about acute abdominal pain that started 20 min just after vaginal intercourse. Physical examination revealed marked pallor, abdominal tenderness without uterine rigidity and vaginal bleeding. The cervix was long and posterior. The patient denies any history of direct trauma or domestic violence and there was not any sign of trauma on the clinical exam. Her blood pressure was 90/50 mmHg, the pulse rate 68 beats per minute (bpm). The nonstress test (NST) revealed a normal fetal heart rate (145/min) with moderate variability and no uterine contractions were detected. The patient responded briefly to the rapid infusion of 1 l of normal saline before she collapsed and re-experienced hypotension (80/50). A bedside ultrasound, that was performed in the labor room, revealed a hemoperitoneum and fluid in the Morison pouch. The NST showed a prolonged deceleration for 5 min. An emergent caesarean delivery was decided.

Laboratory results showed hemoglobin of 8.4 g/l; platelets, coagulation panel, fibrinogenemia, liver function test, and lipase, were all normal.

Pfannenstiel incision was done under spinal anesthesia. Intra-abdominal adhesions caused by previous cesarean sections were noted. A large hemoperitoneum containing more than 2 l of fresh blood and clots was noted. A transverse incision on the lower uterine segment was done and the baby was extracted. We did not identify any abnormality of the uterus or the placenta neither a retroplacental hematoma. The uterine veins and adnexa were normal. However, the bleeding continued and we remark that it was coming from the upper abdomen.

**Figure 1.**
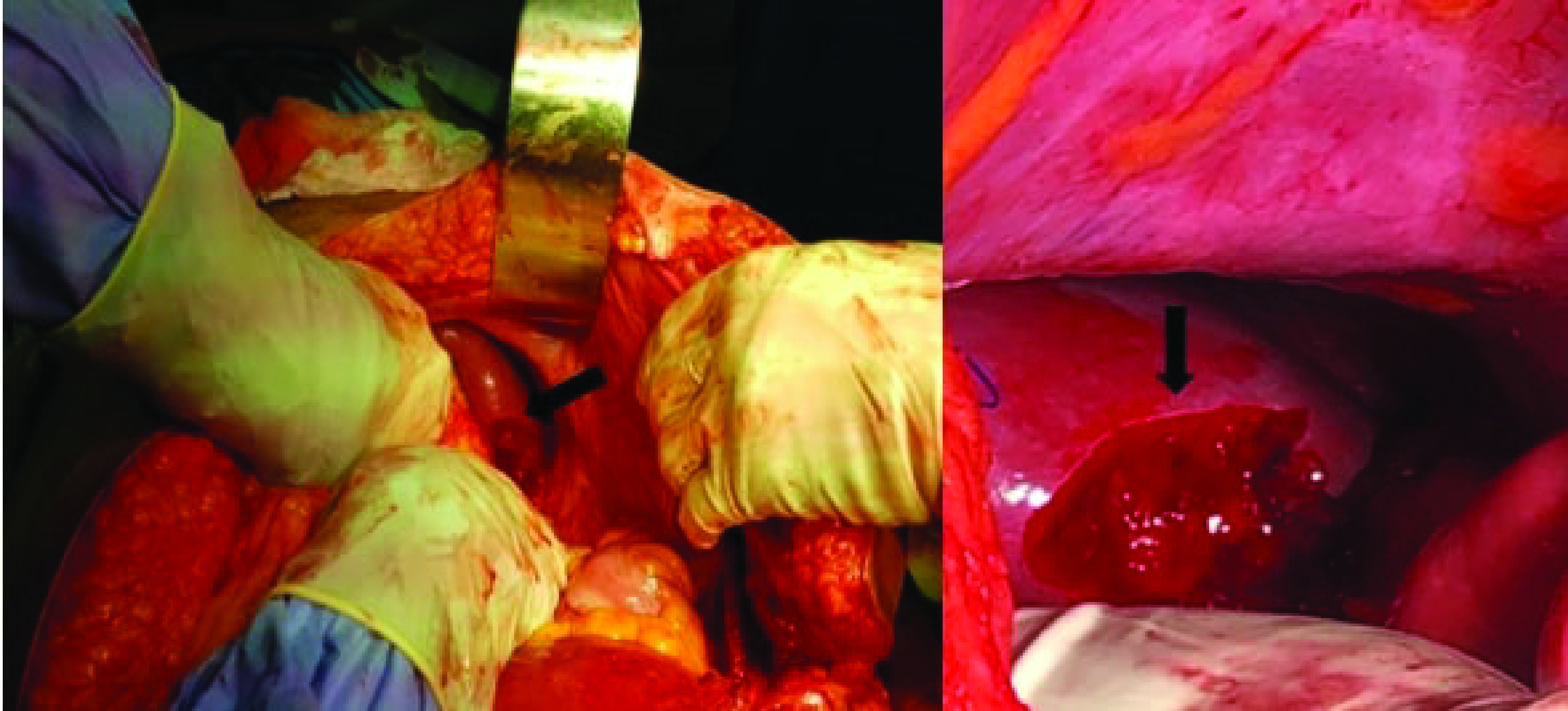
Splenic rupture at the splenocolic ligament insertion (arrow).

The abdominal incision was extended vertically along with general anesthesia. Exploration showed intra-abdominal adhesions and a double tear in the spleen was seen on the splenocolic and gastrosplenic ligament insertions showing active bleeding (Figure 1). Conservative treatment of the spleen was performed by a general surgeon using hemostatic sutures and compression. During the surgery, the patient received a total of seven units (three packed red blood cells, four fresh frozen plasma). Her post operative hemoglobin was 7 g/l and she received two additional packed red blood cells. The patient's postoperative course was uneventful, and she was discharged home on day 5.

Of note, the decision-to-delivery interval was 20 min during which the fetal heart rate was normal (category I) except for the last 5 min of deceleration, just before the transfer to the operating room.

When delivered, the newborn presented an Apgar score = 0. The infant was resuscitated at birth and then intubated. Apgar scores were 0/3/4 at 0/5/10 min, respectively. He was admitted to the neonatal intensive care unit (NICU) where umbilical cord blood gas showed severe acidemia (pH: 6.68). His hospitalization was complicated by a resolutive episode of acute renal failure. Cultures were negative. He was extubated on day 4 and feeding started progressively with good swallowing reflex. After six months, the baby presented a normal neurological development in his follow-up visit.

An electroencephalogram was done and was not conclusive. He was discharged on day 18.

## Discussion

We reported a case of traumatic postcoital spleen rupture at 36 weeks of pregnancy leading to maternal and neonatal morbidity.

Sexual activity is generally considered safe in pregnancy with a tendency to decrease in frequency with advancing gestational age [6,7], except for women at risk of preterm labor or placenta previa. This decrease is in part attributable to fear of harming the fetus, fear of membrane rupture, or fear of infection [8].

Among cases of spleen rupture during pregnancy, sexual intercourse is an extremely rare cause and only one case was reported in Sparkman's review of literature in 1958 [4].

Table 1 shows a comparison with the only reported similar case of spleen rupture after sexual intercourse.

**Table 1.** Comparison of characteristics of spleen ruptures cases with sexual intercourse.

	Stretton's case reported by Sparkman	Our case
Maternal age (years)	37	33
Term of pregnancy	5th month (week not specified)	36 weeks
Interval coitus-symptoms	2–3 h	20 min
Symptoms	Abdominal pain	Abdominal pain-recurrent collapse
Treatment	Splenectomy in 12 h	C-section splenorrhaphy in 40 min
Surgical exploration	Not described	Double tear in the colosplenic and gastrosplenic ligament insertion
Pregnancy outcome	Not described	Fetus delivered in cardiac arrest, resuscitated, discharged 18 days later
Maternal outcome	Lived	Lived

Data taken from [2].

**Figure 2.**
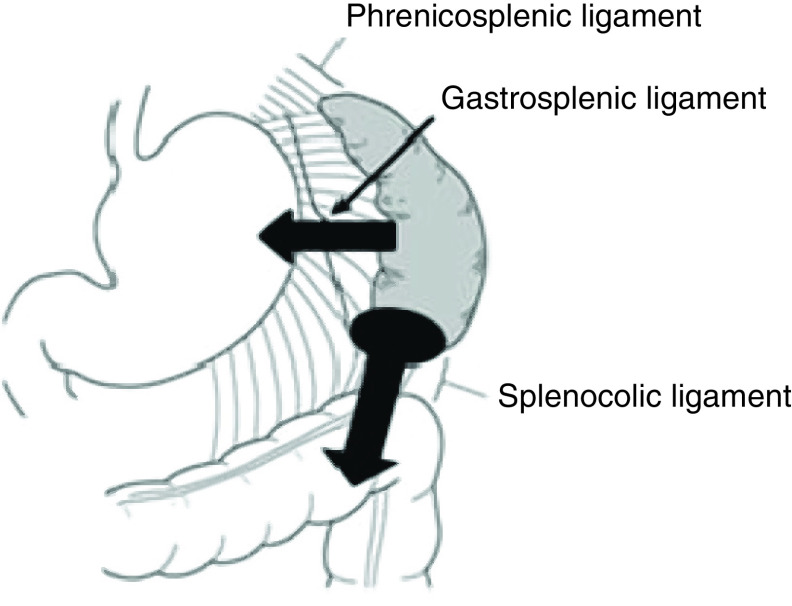
The mechanism of splenic rupture in our case. Arrows: traction on the splenocolic and gastrosplenic ligaments.

The majority of reported cases of spleen rupture during pregnancy occurred in the third trimester and in a multiparous woman which is similar to our case [4,5]. One of the suggested mechanisms of splenic rupture explains that following a trauma, the ligamentous suspension of the spleen limits its motion to a certain degree, then fixation occurs followed by laceration or avulsion [5]. Besides, intra-abdominal adhesions caused by previous caesarian sections would increase the traction on splenocolic ligament during intercourse and causes avulsion of the splenic capsule [9]. Figure 2 shows the ligamentous suspension of the spleen and explains the hypothesized mechanism of splenic rupture in our case.

This case occurred in the absence of pre-existing pathology of the spleen and signs of abdominal trauma. Surgical exploration showed the presence of a double tear on the splenocolic and gastrosplenic ligaments insertions at the spleen (Figure 1) along with the history of abdominal pain that started after vaginal intercourse. Based on this, we would classify this case as a traumatic/post-coital rupture of the spleen.

The emergent decision of the cesarean section was based on the acute fetal distress. The origin of the intra-peritoneal fluid was inexplicable. The heart rate (68 bpm) was not in favor of hemorrhagic shock. The absence of vaginal bleeding, the absence of labor, the normal uterine tonus and normal NST in the first were not in favor of uterine rupture or abruptio placenta.

The diagnosis of splenic rupture may be difficult due to the poor clinical presentation and signs, especially when no evidence of trauma or predisposing factors. In fact, the clinical presentation of a splenic rupture can be insidious: a falsely reassuring stable blood pressure while a hemoperitoneum is being grown slowly, then signs of shock and collapse occur [10]. Thus, based on these clinical presentations, a cesarean section with spinal anesthesia and Pfannenstiel incision was performed, and the decision of the conversion into general anesthesia with vertical incision was taken just after detecting an upper origin of the bleeding. Although the ultrasound provides high sensitivity and specificity for detecting major abdominal trauma in pregnancy [11], the rarity of this presentation led us to the ignorance of bleeding origin and to explore it during the surgery.

## Conclusion

Traumatic spleen rupture is an etiology to consider in pregnant women presenting with acute abdominal pain following sexual intercourse. Early suspicion and emergent surgical management are the keys to optimize maternal and perinatal outcomes. Adjunction of abdominal ultrasound can hasten the diagnosis and urge surgical intervention. Stable blood pressure and reassuring fetal heart rate should not delay surgery. Such a delay would increase maternal and neonatal morbidity.

## Future perspective

This case report highlights the importance of ultrasound in the labor ward. Based on our experience, we support workshops and hands-on training in ultrasound among medical students, midwives, and emergency physicians in order to improve our practice. Moreover, further studies are necessary to better understand the safety of sexual intercourse in the third trimester among pregnant women.

**Executive summary**

**Background**

- Rupture of the spleen is a serious emergency that occurred after a blunt trauma of the abdomen or after an evolution of a splenomegaly.
- Rupture of the spleen is an unusual but serious cause of acute abdominal pain in pregnancy and is associated with both maternal and fetal morbidity.

**Case report**

- A 33-year-old pregnant woman presented at 36 weeks gestation to the emergency with acute abdominal pain that started after vaginal intercourse.
- No bruising was present on the abdominal examination.
- A bedside ultrasound, that was performed in the labor room, revealed a hemoperitoneum and fluid in the Morison pouch.
- An emergent cesarean delivery was performed for resistant hypotension and collapse.
- Active spleen bleeding was identified at the splenocolic and gastrosplenic ligament insertion.
- The patient had a conservative treatment of the spleen and an uncomplicated postoperative course.

**Discussion**

- One of the suggested mechanisms of splenic rupture explains that following a trauma, the ligamentous suspension of the spleen limits its motion to a certain degree, then fixation occurs followed by laceration or avulsion.

**Conclusion**

- Traumatic spleen rupture is an etiology to consider in pregnant women presenting with acute abdominal pain following sexual intercourse.
- Early suspicion and emergent cesarean delivery are the keys to optimize maternal and perinatal outcomes.

**Future perspective**

- Workshops and hands-on training in ultrasound among medical students, midwives, and emergency physicians are necessary to improve our practice.
